# Target Uncertainty Mediates Sensorimotor Error Correction

**DOI:** 10.1371/journal.pone.0170466

**Published:** 2017-01-27

**Authors:** Luigi Acerbi, Sethu Vijayakumar, Daniel M. Wolpert

**Affiliations:** 1 Institute of Perception, Action and Behaviour, School of Informatics, University of Edinburgh, Edinburgh, United Kingdom; 2 Doctoral Training Centre in Neuroinformatics and Computational Neuroscience, School of Informatics, University of Edinburgh, Edinburgh, United Kingdom; 3 Computational and Biological Learning Lab, Department of Engineering, University of Cambridge, Cambridge, United Kingdom; University of Exeter, UNITED KINGDOM

## Abstract

Human movements are prone to errors that arise from inaccuracies in both our perceptual processing and execution of motor commands. We can reduce such errors by both improving our estimates of the state of the world and through online error correction of the ongoing action. Two prominent frameworks that explain how humans solve these problems are Bayesian estimation and stochastic optimal feedback control. Here we examine the interaction between estimation and control by asking if uncertainty in estimates affects how subjects correct for errors that may arise during the movement. Unbeknownst to participants, we randomly shifted the visual feedback of their finger position as they reached to indicate the center of mass of an object. Even though participants were given ample time to compensate for this perturbation, they only fully corrected for the induced error on trials with low uncertainty about center of mass, with correction only partial in trials involving more uncertainty. The analysis of subjects’ scores revealed that participants corrected for errors just enough to avoid significant decrease in their overall scores, in agreement with the minimal intervention principle of optimal feedback control. We explain this behavior with a term in the loss function that accounts for the additional effort of adjusting one’s response. By suggesting that subjects’ decision uncertainty, as reflected in their posterior distribution, is a major factor in determining how their sensorimotor system responds to error, our findings support theoretical models in which the decision making and control processes are fully integrated.

## Introduction

Sensorimotor tasks typically involve both estimating the state of the world (e.g., target and limb positions) and controlling actions so as to achieve goals. Two major frameworks, Bayesian estimation and stochastic optimal feedback control (OFC), have emerged to explain how the sensorimotor system estimates uncertain states and controls its actions. Together these frameworks have provided a normative account of human motor coordination which is able to account for a range of behavioral phenomena, including how humans correct for perturbations of various kind in fast directed movements. Here we will investigate the relation between estimation and online control.

Estimation is a nontrivial task due to sensory noise [1] and the ambiguity of the stimuli [2]. Optimal estimates need to take into account the statistics of the stimuli, the currently available information, and the cost associated with errors in the estimate [3, 4]. Humans have been shown to combine prior information with sensory data in a manner broadly consistent with Bayesian integration in a variety of sensorimotor tasks, such as reaching [5], interval timing [6, 7], pointing to hidden targets [8–10], speed estimation [11, 12], orientation estimation [13], and motion estimation [14]. Humans are also sensitive to the reward/loss structure imposed by the task [15]. Within the framework of Bayesian Decision Theory (BDT), this means that probabilistic ‘posterior’ estimates are combined with a cost function so as to maximize the expected gain [16]. Estimation performance compatible with BDT, with an explicitly imposed loss function, has been observed, for example, in visual ‘offset’ estimation [17], orientation estimation [18], motor planning [19], and sensorimotor timing [7]. These studies suggest that people keep track of the uncertainty, and possibly build a full probabilistic representation [20], of perceptual and sensorimotor variables of interest, and use it to compute optimal estimates.

Optimal feedback control (OFC) is a prominent theory of motor control whereby optimal feedback gains are computed by minimizing the cost of movement over the space of all possible feedback control strategies [21–23]. The ability of the sensorimotor system to make online corrections in OFC is crucial in the presence of errors that can arise from both the inaccuracies in internal models that are involved in generating the commands [24, 25] and from the noise and variability inherent in sensory inputs and motor outputs [1, 26]. The cost function in OFC takes into account various factors, with a trade-off between task goals (accuracy) and effort (energy, movement time, computation); see, e.g., [27]. A prediction of OFC is the minimal intervention principle, according to which errors are corrected and movement variability is minimized only along task-relevant dimensions [21]. OFC also suggests how the motor system should react to perturbations throughout the movement. For example, for fast directed reaching, late perturbations afford a lesser correction gain due to a trade-off between accuracy and stability [28]. A few studies have investigated online control of movement in the presence of uncertain targets, finding agreement with the optimal solution given by a Kalman filter, which describes iterative Bayesian integration of sensory information according to its reliability [29–31]. Recent work on the interaction between uncertainty and control has also found that human sensorimotor behavior exhibits risk-sensitivity, that is sensitivity to the uncertainty in the reward [32, 33], which may stem from target variability [34]. In sum, there are both theoretical and empirical reasons to suggest that uncertainty in the estimate may interfere with the way in which humans correct online for their sensorimotor errors.

Online error correction during reaching has typically been studied by observing how subjects react to either mechanical perturbations or explicit or subliminal alterations of visual feedback of the hand (e.g., [35, 36]) or of the target (see [37] for a review). Measured correction gains have been shown to change across the movement [38] and according to task demands, in agreement with OFC [28]. Also, as mentioned before, subjects do not correct indiscriminately for all perturbations, but mostly only for those along task-relevant dimensions [21]. For example, a recent study has shown that subjects used a flexible control strategy that adapted to task demands (target shape) according to the minimal intervention principle on a trial-by-trial basis [39]. These studies, however, mostly examine feedback control in the presence of well-defined, visible targets and with fast movements (duration under 1 second). Here we investigate the relation between estimation and control, by asking if uncertainty in the estimation influences the process of online error correction even when sufficient time is available to fully correct for any errors, here represented by a late visual perturbation.

In our experiment, subjects performed a center of mass estimation task in which they were presented with a visual dumbbell (a bar with disks on each end) and were required to place their finger on the bar to balance the dumbbell. The task was designed so that the estimation would have low variability on some trials (same size disks requiring a simple line bisection to balance) or high variability (unequal sized disks). During the reaching movement to indicate the balance point the location of the finger was occluded and on some trials, unbeknownst to participants, when the finger reappeared its position had been visually shifted so that we could then examine the extent to which subjects corrected for the shift.

We can consider three scenarios. If subjects estimate the center of mass position as a point estimate and then simply report this with a reach, then we would expect that they should correct for the entire perturbation to be as accurate as possible—or, if there is a cost of correction, they should correct just as much for the high and low uncertainty conditions. If subjects represent the full posterior of the position but have no cost on corrections then we would expect that they should correct for the entire perturbation to be as accurate as possible. However, if subjects represent their uncertainty in the center of mass location, as reflected in their posterior distribution, they may be less willing to correct in the high-uncertainty condition as the cost of correction (e.g., energy, movement time, computation) may outweigh the potential increases in accuracy that can be achieved through correction.

Even though participants were given enough time to compensate for the perturbation, they only fully corrected for the induced error on trials with low uncertainty about target location and corrected partially in conditions with more uncertainty (where partial correction was just enough to make their performance practically indistinguishable from the unperturbed trials). Our findings suggest that subjects’ decision uncertainty, as reflected in the width of the posterior, is a factor in determining how their sensorimotor system responds to errors, providing new evidence for the link between decision making and control processes.

## Materials and Methods

### Participants

Sixteen naïve subjects (8 male and 8 female; age range 19–27 years) participated in the study. All participants were right-handed [40], with normal or corrected-to-normal vision and reported no neurological disorder. The Cambridge Psychology Research Ethics Committee approved the experimental procedures and all subjects gave written informed consent.

### Behavioral task

**Fig 1:**
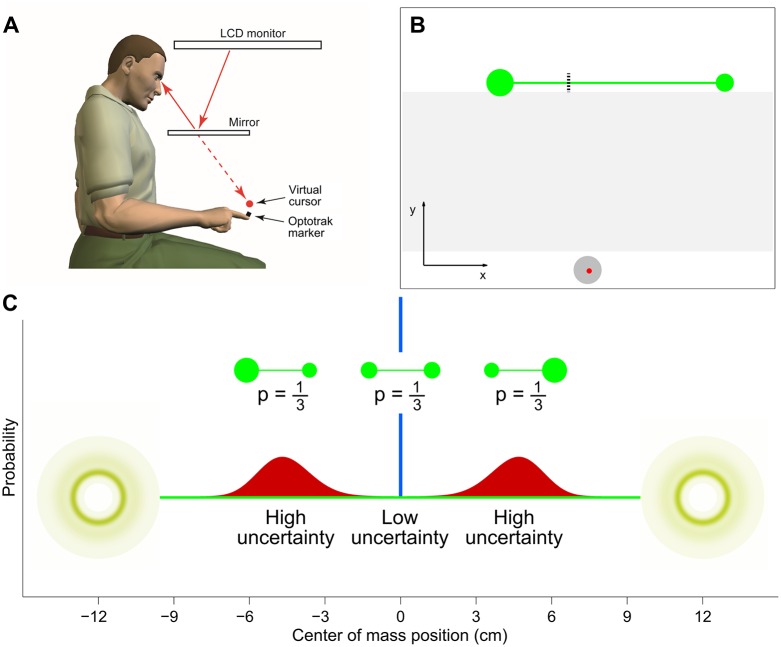
Experimental setup. *A:* Subjects wore an Optotrak marker on the tip of their right index finger. The visual scene from a CRT monitor, including a virtual cursor that tracked the finger position, was projected into the plane of the hand via a mirror. *B:* The screen showed a home position at the bottom (grey circle), the cursor (red circle), here at the start of a trial, and the object at top (green dumbbell). The task consisted of locating the center of mass of the object, here indicated by the dashed line. Visual feedback of the cursor was removed in the region between the home position and the target line (here shaded for visualization purposes). *C:* The two disks were separated by 24 cm and, depending on the disks size ratio, the target (center of mass) was either exactly halfway between the two disks (*p* = 1/3; low uncertainty; blue distribution) or to the right (*p* = 1/3) or left (*p* = 1/3) of the midpoint (high uncertainty; red distributions), leading to a trimodal distribution of center of mass.

Subjects performed a center of mass estimation task, designed to probe subject’s behavior in a natural sensorimotor task. We used an Optotrak 3020 (Northern Digital Inc, Ontario, Canada) to track the tip of a subject’s right index finger at 500 Hz. The visual image from a LCD monitor (Apple Cinema HD, 64 cm × 40 cm, 60 Hz refresh rate) was projected into the plane of the hand via a mirror that prevented the subjects from seeing their arm (Fig 1A). The workspace origin, coordinates (0, 0), was ∼20 cm in front of the subject’s torso in the mid-sagittal plane, with positive axes towards the right (‘horizontal’ *x* axis) and away from the subject (‘vertical’ *y* axis). The workspace showed a home position (1.5 cm radius circle) at the origin and a cursor (0.25 cm radius circle) could be displayed that tracked the finger position.

On each trial a virtual object consisting of two filled circles (disks) and a thin horizontal line (*target line*) connecting the centers of the two disks [41] was displayed on the screen (Fig 1B). The centers of the disks were *ℓ* = 24 cm apart (length of the target line) and at vertical position *y* = 20 cm. To prevent subjects from responding to a stereotypical location in the workspace, on each trial the object was horizontally displaced with a uniformly random jitter ∼[−3, 3] cm from the center of the screen. The radius of one of the disks was drawn from a log-normal distribution with mean log 1 cm and SD 0.1 in log space. The radius of the other disk was chosen so that on 1/3 of the trials the disks were of equal size, making the task equivalent to a simple line bisection, and on 2/3 of the trials the ratio of the disk radii was drawn from a log-normal distribution with mean log 1.5 and SD 0.1 in log space, leading to a trimodal distribution of center of mass locations (Fig 1C). The bulk of the distribution over locations was far (≫2 cm) from the largest disks’ edges, so as to avoid edge effects [41]. The position (left or right) of the larger disk in unequal-size trials was chosen randomly and counterbalanced within each experimental block. We expected that the uncertainty in the center of mass location would be low for the equal-disk trials (‘Low-uncertainty’), when the task was equivalent to line bisection, but would be high for the unequal-disk trials (‘High-uncertainty’) due to both the spread of the experimental distribution and the nonlinear mapping between the disks’ ratio and center of mass, see below.

After a ‘go’ tone, participants were required to reach from the home position to the center of mass of the disks (the *target*) on the target line, thereby balancing the object on their finger. Subjects were explicitly told in the instructions that the circles were to be interpreted as disks in the center of mass estimation. Importantly, during the reaching movement, visual feedback of the cursor was removed in the region *y* ∈ [2, 19] cm (shaded area in Fig 1B). Subjects were given 1.5 s to arrive in the proximity of the target line (*y* > 19.5 cm). After reaching the target line, subjects were allowed 3 seconds to adjust their endpoint position to correct for any errors that might have arisen during the movement when the cursor was hidden. The remaining time for adjustment was indicated by a pie-chart animation of the cursor, which gradually turned from red to yellow. The cursor’s horizontal position at the end of the adjustment phase constituted the subject’s response for that trial. If participants were still moving at the end of the adjustment phase (velocity of the finger greater than 0.5 cm/s), the trial was terminated with an error message. Such missed trials were presented again later during the session.

### Experimental sessions

Participants performed a preliminary training session (120 trials) in which they received performance feedback at the end of each trial. Performance feedback consisted of displaying the correct location of the center of mass, an integer score and, if the error was greater than 1 cm, a tilted dumbbell in the appropriate direction. The score depended on the (horizontal) distance of the cursor from the center of mass, Δ*s*, according to a squared exponential formula:

$$\begin{array}{c} \text{Score}\left(\Delta s\right)=\text{Round}(10\cdot \exp\{-\frac{\Delta s^{2}}{2\sigma_{\text{score}}^{2}}\}), \end{array}$$ (1)

where *σ*_score_ is the score length scale and Round(*z*) denotes the value of *z* rounded to the nearest integer. We chose the numerical constants in Eq (1) (*σ*_*Score*_ ≈ 0.41 cm) such that the score had a maximum of 10 and was nonzero up to 1 cm away from the center of mass. A new trial started 500 ms after the subject had returned to the home position.

Subjects then performed a test session (576 trials) which included standard trials (192 trials) identical to the training session, and ‘perturbation’ trials in which, unbeknownst to the subjects, the visual feedback of the cursor was displaced horizontally from the finger when the cursor reappeared at the end of the movement (*y* > 19 cm), near the target line. Cursor displacement could either be small (drawn from a Gaussian distribution with mean ±0.5 cm and SD 0.2 cm; 192 trials), or large (mean ±1.5 cm and SD 0.2 cm; 192 trials). To avoid overlap between distinct perturbation levels, the Gaussian distributions were truncated at 2.5 SDs (0.5 cm away from the mean). All trials were presented in a pseudorandom order and left and right perturbations were counterbalanced within the session. To keep subjects motivated throughout the test session while minimizing the chances that subjects would either adapt their behavior or become aware of the shifts, we only provided participants with performance feedback on unperturbed trials [5]. We also provided the sum of the scores for all trials in blocks of 36 trials [17]. All participants answered a short debriefing questionnaire at the end of the session, the results of which showed that they were unaware of the perturbations or of any other difference between trials with or without performance feedback (see S1 Appendix for details).

### Data analysis

For all analyses the criterion for statistical significance was *p* < .05, and we report uncorrected *p*-values. Even after applying a conservative Bonferroni correction for multiple comparisons with *m* = 20 (for the about twenty different analyses we conducted) all of our main findings remain statistically significant. Unless specified otherwise, summary statistics are reported in the text as mean ± SE between subjects.

### Trial response data

For each trial, we recorded the final horizontal position *r* of the visual cursor, the horizontal position of the hidden cursor at the time of exiting the no visual feedback zone *x*_exit_, and the effective adjustment time (time before the subject stopped moving during the adjustment phase). We computed the response error Δ*s* as the signed difference between the final position of the visual cursor and position of the center of mass of the current stimulus.

### Variation of mean residual error and SD of the error

We analyzed how the mean residual error (or ‘bias’) and SD of the error depended on the class of stimuli presented (Low-uncertainty and High-uncertainty) and on the mean perturbation level (−1.5, −0.5, 0, 0.5, 1.5). For the High-uncertainty trials we had counterbalanced whether the larger disk was on the right or left. An examination of the mean residual error and SD of the error with factor of side (Left, Right) and perturbation mean level showed no significant difference and we therefore pooled data from Left trials with Right trials.

Statistical differences between conditions in these analyses were assessed using repeated-measures ANOVA (rm-ANOVA) with Greenhouse-Geisser correction of the degrees of freedom in order to account for deviations from sphericity [42]. A logarithmic transformation was applied to the SDs before performing rm-ANOVA, in order to improve normality of the data (results were qualitatively similar for non-transformed data). We report effect sizes as partial eta squared, denoted with $\eta_{\text{p}}^{2}$.

### Slope of the mean residual error

For each subject, we performed linear regression of the mean residual error as a function of perturbation size (a continuous variable from −2 to 2 cm) for the Low and High uncertainty conditions. The slope of the regression fit is a measure of the fraction of the applied perturbation that was not corrected for. In the plots, we remove the mean residual error for the 0 perturbation condition from each subject’s data to allow a direct comparison between subjects; this has no effect on the estimation of the slope. The difference in slope between conditions was assessed with a paired Student’s *t*-test on the individual slope coefficients.

### Observer model

We built a Bayesian observer model to investigate whether our subjects’ correction biases could be explained as the interaction of probabilistic inference and the correction cost. In order to account for the residual errors (lack of correction) in the perturbation condition, we introduced a modification to the structure of the loss function that takes *effort* into account. As described below, subjects’ datasets were fit individually and model fits were averaged to obtain the group prediction. To limit model complexity and avoid overfitting, some model parameters were either estimated from the individual training datasets or fixed to theoretically motivated values.

### Perception stage

We assume that the observer estimates the log ratio of the radii of the two disks, whose true value is *ρ* = log(*r*_2_/*r*_1_), where *r*_*i*_ with *i* = 1, 2 is the radius of the two disks (left and right) presented on a trial. This logarithmic representation was chosen as it naturally embodies Weber’s law. It also unifies different possible transformations of radius to weight for each disk that the subject might use. For example if subjects use the radius to calculate the area or volume (as though the object was a sphere) then the log ratio can be simply expressed as $\log(r_{2}^{2}/r_{1}^{2})=2\rho$ and $\log(r_{2}^{3}/r_{1}^{3})=3\rho$, respectively.

In the estimation process, the true ratio is corrupted by normally distributed noise with magnitude *σ*_*ρ*_ in log space, which yields a noisy measurement *ρ*_*m*_. The parameter *σ*_*ρ*_ represents both log-normally distributed sensory noise in estimating the radii of the disks and additional independent sources of error in computing the ratio (see Discussion). The conditional measurement probability takes the form:

$$\begin{array}{c} p_{\text{meas}}\left(\rho_{m}|\rho \right)=N(\rho_{m}|\rho ,\sigma_{\rho }^{2}), \end{array}$$ (2)

where $N(x|\mu ,\sigma^{2})$ is a normal distribution with mean *μ* and variance *σ*^2^.

The experimental distribution of log ratios is a mixture of three components: two Gaussians centered at ±log 1.5 ≈ ±0.405 with SD 0.1 and a delta function at *ρ* = 0 (Fig 1B). For simplicity, we assume the observer’s prior in log-ratios space, *q*_*prior*_(*s*), corresponds to the experimental distribution:

$$\begin{array}{c} q_{\text{prior}}\left(\rho \right)=\frac{1}{3}\sum_{i=1}^{3}N(\rho |\mu_{\text{prior}}^{\left(i\right)},{\sigma_{\text{prior}}^{\left(i\right)}}^{2}), \end{array}$$ (3)

with ***μ***_prior_ = (−log 1.5, 0, log 1.5) and ***σ***_prior_ = (0.1, 0, 0.1), using the formal definition N(x|μ,0)≡δ(x-μ).

Combining Eqs (2) and (3), after some algebraic manipulations, the posterior can be expressed as a mixture of Gaussians [10]:

$$\begin{array}{c} q_{\text{post}}\left(\rho |\rho_{m}\right)=\frac{1}{Z}\sum_{i=1}^{3}Z^{\left(i\right)}N(\rho |\mu_{\text{post}}^{\left(i\right)},{\sigma_{\text{post}}^{\left(i\right)}}^{2}), \end{array}$$ (4)

where the normalization factor Z, the posterior mixing weights, means, and variances have all a closed-form expression (see S1 Appendix).

The observer uses the inferred values of *ρ* to compute the location of the center of mass of the two-disk object (here measured with respect to the midpoint between the two disks). We denote with *f*_*D*_(*ρ*) the generally nonlinear mapping that identifies the location of the center of mass *s* of two *D*-dimensional spheres with radii of log ratio *ρ* (see S1 Appendix). We assume that the observer computes the center of mass using this mapping *f*_*D*_ with some fixed value of *D* > 0, although not necessarily the correct value *D* = 2 for two-dimensional disks, nor we restrict *D* to be an integer. Knowing the expression for *f*_*D*_(*ρ*), we can compute the posterior distribution of the location of the estimated center of mass, *q*_post_(*s*|*ρ*_*m*_) (see S1 Appendix for the derivation). Due to the generally nonlinear form of *f*_*D*_, this posterior is a mixture of non-Gaussian distributions. However, we find that it is well approximated by a mixture of Gaussians:

$$\begin{array}{c} q_{\text{post}}\left(s|\rho_{m}\right)\approx \frac{1}{Z}\sum_{i=1}^{3}Z^{\left(i\right)}N(s|m_{\text{post}}^{\left(i\right)},{s_{\text{post}}^{\left(i\right)}}^{2}), \end{array}$$ (5)

where $m_{\text{post}}^{\left(i\right)}$ and $s_{\text{post}}^{\left(i\right)}$ are respectively the mean and SD of the mixture components of the posterior (see S1 Appendix for details).

### Decision-making stage

According to Bayesian Decision Theory (BDT), the observer chooses the final cursor position that minimizes his or her expected loss [16]. The typical loss functions used in perceptual and even sensorimotor tasks take into account only the error (distance between response and target). However, although the explicit goal of our task consists of minimizing endpoint error, subjects appeared to be influenced by other considerations.

We assume that the subjects’ full loss function depends on an error-dependent cost term, $L_{\text{err}}\left(r-s\right)$, which assesses the deviation of the response (*r*) from the target (*s*), and a second adjustment cost, $L_{\text{adj}}\left(r-r_{0}\right)$, which expresses the cost of moving from the perturbed endpoint position *r*_0_ (originally planned endpoint position plus perturbation *b*). The rationale is that there is an additional cost in moving from the initially planned endpoint position, possibly due to the effort involved in an additional unplanned movement (e.g., for replanning the action).

In a preliminary motor planning stage, the endpoint $s_{\text{pre}}^{*}$ is chosen by minimizing the error loss:

$$\begin{array}{ccc} s_{\text{pre}}^{*}\left(\rho_{m}\right) & = & \arg\min_{\hat{s}}[\int_{-\ell /2}^{\ell /2}q_{\text{post}}\left(s|\rho_{m}\right)L_{\text{err}}(\hat{s}-s)ds]\\ & = & \arg\min_{\hat{s}}[-\sum_{i=1}^{3}Z^{\left(i\right)}N(\hat{s}|m_{\text{post}}^{\left(i\right)},{s_{\text{post}}^{\left(i\right)}}^{2}+\sigma_{\text{err}}^{2})], \end{array}$$ (6)

where we assumed for the loss function a continuous approximation of the discrete scoring system (Eq (1)), that is a (rescaled) inverted Gaussian, $L_{\text{err}}\left(\hat{s}-s\right)=-\exp\{-{\left(\hat{s}-s\right)}^{2}/2\sigma_{\text{err}}^{2}\}$. In addition to being both in agreement with the reward structure of the task and a loss that well describes human sensorimotor behavior [43], this loss allowed us to derive an analytic solution for the expected loss (Eq (6)). To limit model complexity, we assumed subjects conformed to the error length scale of the performance feedback, that is *σ*_err_ = *σ*_score_ (Eq (1)).

After the initial movement, subjects are allowed plenty of time to adjust their endpoint position. Due to the applied perturbation *b*, the (average) endpoint position after movement will be $r_{0}\equiv s_{\text{pre}}^{*}\left(\rho_{m}\right)+b$. We introduce, therefore, the adjustment cost in the final loss function:

$$\begin{array}{c} L(r,s,r_{0})=L_{\text{err}}\left(r-s\right)+\alpha L_{\text{adj}}\left(r-r_{0}\right), \end{array}$$ (7)

where *α* ≥ 0 specifies the relative weight of the adjustment loss with respect to the error term. In Eq (7), *r*_0_ represents the (average) end point before adjustment and *r* the endpoint after adjustment, so that the adjustment loss is a function of the distance covered in the adjustment phase, *r* − *r*_0_. The key characteristic of this loss function is that for Low-uncertainty trials the first term can be significantly reduced by adjustments, whereas for High-uncertainty trials there is less to be gained through adjustments (as the location of the center of mass has high variance) and the second term can become dominant leading to partial correction, with *α* controlling this trade-off. The ‘optimal’ final position s* that minimizes the expected loss in Eq (7) is:

$$\begin{array}{ccc} s^{*}\left(\rho_{m},r_{0}\right) & = & \arg\min_{\hat{s}}[\alpha L_{\text{adj}}\left(\hat{s}-r_{0}\right)+\int_{-\ell /2}^{\ell /2}q_{\text{post}}\left(s|\rho_{m}\right)L_{\text{err}}(\hat{s}-s)ds]. \end{array}$$ (8)

For simplicity, for $L_{\text{adj}}\left(\hat{s}-r_{0}\right)$ we also assume the shape of an inverted Gaussian loss with length scale *σ*_adj_, a free parameter of the model representing the scale of the cost of moving away from the originally planned target. For the chosen loss functions, Eq (8) can be further simplified (see S1 Appendix for details), but still only admits numerical solution. In section ‘Alternative observer models’, we will see how the solution of Eq (8) changes depending on the shape of the loss functions.

### Full observer model

In each trial, the decision-making process is simulated in two stages. First, the observer computes the preliminary endpoint position $s_{\text{pre}}^{*}\left(\rho_{m}\right)$ for a given internal measurement *ρ*_*m*_ (Eq (6)). For simplicity, we assume that the endpoint position is systematically altered only by the external perturbation *b*, so that (on average) the arrival position is $r_{0}=s_{\text{pre}}^{*}\left(\rho_{m}\right)+b$. In the second step, the observer adjusts his or her endpoint position, moving to the optimal target as per Eq (8). Gaussian noise with variance $\sigma_{\text{motor}}^{2}$ is added to the final choice s* to simulate any residual noise in the response.

According to this model, the response probability of observing response *r* in a trial with perturbation *b* and disks’ ratio *ρ* is:

$$\begin{array}{c} \mathrm{Pr}(r|\rho ,b;\theta )=\int_{-\infty }^{\infty }N(\rho_{m}|\rho ,\sigma_{\rho }^{2})N(r|s^{*}\left(\rho_{m},x_{\text{pre}}^{*}\left(\rho_{m}\right)+b\right),\sigma_{\text{motor}}^{2})d\rho_{m}, \end{array}$$ (9)

where we marginalized over the internal measurement *ρ*_*m*_ which is not directly accessible in our experiment, and ***θ*** = {*σ*_*ρ*_, *D*, *α*, *σ*_adj_, *σ*_motor_} is the vector of model parameters.

We estimated the model parameters for each subject via maximum-likelihood (see S1 Appendix for details). To limit the possibility of overfitting, the sensory variability parameter of each subject, *σ*_*ρ*_, was estimated from a separate model fit of the training datasets. The observer model fit to the individual test datasets had, therefore, effectively 3 free parameters: *D*, *α* and *σ*_adj_ representing the dimensionality of the transformation from disk radius to weight, the trade-off between error and effort and the length-scale of the loss function for adjustments, respectively. The parameter $\sigma_{\text{motor}}^{2}$ represents the mean square of the residuals and is not typically counted as a free parameter.

## Results

### Human performance

Subjects found the task natural and straightforward to perform and the debriefing questionnaire at the end of the session showed that they were unaware of the perturbations on the trials. On unperturbed Low-uncertainty trials they received on average 7.36 ± 0.43 points and balanced the object on 97.4% of trials. In contrast on High-uncertainty trials they received on average 3.35 ± 0.15 points and balanced the object on 60.2% of trails. Example subject trajectories and velocity profiles are shown in S1 Fig.

### Mean residual error and variability

We analyzed the participants’ response (visual location of cursor at the end of the adjustment phase) as a function of trial uncertainty (Low, High) and mean perturbation level (−1.5, −0.5, 0, 0.5, 1.5). To confirm that the trials with equal-sized and unequal-sized disks correspond to low and high-uncertainty we examined the variability (SD) of subjects’ response. As expected, we found that the variability was significantly affected only by trial uncertainty (main effect: Low, High; *F*_(1,15)_ = 297, *p* < .001, $\eta_{\text{p}}^{2}=0.94$) with average SD of 0.40 ± 0.06 cm and 1.02 ± 0.05 cm for the Low and High-uncertainty trials, respectively. We found no significant effect of perturbation and no interaction (*p* > .40 and $\eta_{\text{p}}^{2}<0.04$ for both). This confirms that subjects were more variable in their judgments of the center of mass in ‘High-uncertainty’ trials.

We also examined the subjects’ mean residual error (mean difference between cursor endpoint and center of mass). The mean residual error was not significantly affected by trial uncertainty (main effect: Low, High; *F*_(1,15)_ = 0.69, *p* > .40, $\eta_{\text{p}}^{2}=0.04$) but was significantly affected by the perturbation level (main effect: perturbation level; *F*_(3.88,58.1)_ = 25.7, *ϵ* = 0.969, *p* < .001, $\eta_{\text{p}}^{2}=0.63$) and in particular by the interaction between the two (interaction: perturbation × uncertainty; *F*_(3.64,54.7)_ = 15.1, *ϵ* = 0.91, *p* < .001, $\eta_{\text{p}}^{2}=0.50$). This suggests that uncertainty modulates the effect of the perturbation on subjects’ biases.

**Fig 2:**
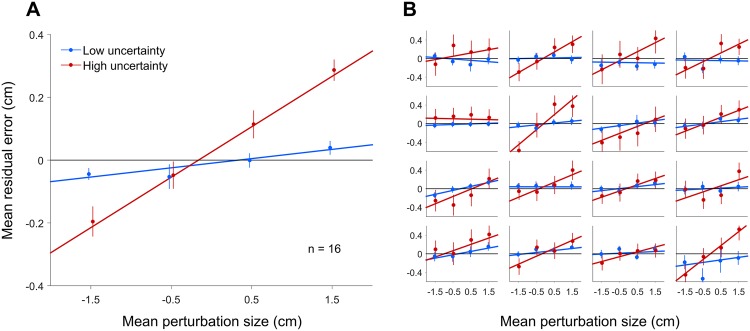
Mean residual error against mean perturbation size, for Low-uncertainty (blue) and High-uncertainty (red) trials. *A:* Group mean residual error against mean perturbation size. Error bars are SEM between subjects. Fits are are linear regressions to the mean data. *B:* Each panel reports the mean residual error against mean perturbation size for a single subject, for Low-uncertainty (blue) and High-uncertainty (red) trials. Error bars are SEM between trials. Fits are linear regressions to the individual data. For both panels each subject’s data have been shifted so as to remove the mean residual error for the 0 perturbation condition for that subject.

To assess the proportion of the perturbation which subjects corrected for, we performed a linear regression of their mean residual error as a function of the perturbation size for Low and High uncertainty trials (after subtracting the baseline mean residual error from unperturbed trials, Fig 2). A slope of zero would correspond to no residual error and hence a full correction, whereas a positive slope correspond to a smaller fraction of the perturbation that subjects correct for, with a slope of 1 corresponding to no correction at all. The regression slopes were small (0.03 ± 0.01) for Low uncertainty trials but large (0.16 ± 0.02) for High uncertainty trials, both significantly different than zero (*t*-test Low: *t*_(15)_ = 3.61, *p* = .003, *d* = 0.90; High: *t*_(15)_ = 8.15, *p* < .001, *d* = 2.04) and significantly different from each other (paired *t*-test *t*_(15)_ = 6.80, *p* < .001, *d* = 1.70). These results show that subjects corrected almost entirely for the perturbation in the Low-uncertainty condition and left sizeable errors in the High-uncertainty trials by only correcting on average for 84% of the perturbation.

### Exit position

On each trial we also recorded the hidden cursor horizontal position when it crossed the end of the no-feedback zone (*y* = 19 cm), before applying visual perturbations, as exit position *x*_exit_. As a sanity check, we verified that subjects’ behavior in perturbation trials *before* applying the perturbation was identical to no-perturbation trials. In particular, we examined the empirical distribution of *x*_exit_ relative to the position of the center of mass for three different levels of perturbation (-1.5, 0, 1.5) and distinct target locations (left, center, and right). The empirical cumulative distribution functions were well overlapping, meaning that indeed there was no systematic difference between perturbation vs. no-perturbation trials.

Then, we examined the variability (SD) of exit position to investigate subjects’ reaching behavior. The SD of *x*_exit_ was respectively 0.89 ± 0.05 cm (Low uncertainty trials) and 1.70 ± 0.07 cm (High uncertainty). We found a statistically significant correlation between the target position and the exit position in the High uncertainty trials (considering Left and Right separately), with a correlation coefficient of *r* = .36 ± 0.02 (*t*-test *t*_(15)_ = 15.0, *p* < .001, *d* = 3.76). Accordingly, the variability of exit position when considered with respect to target position was statistically significantly lower than the variability of *x*_exit_ itself, although not very different in practice (1.64 ± 0.08 cm; paired *t*-test *t*_(15)_ = 3.8, *p* = .002, *d* = 0.96). Also, note that the variability of exit position in Low and High uncertainty trials was substantially higher than the corresponding endpoint variability (*p* < .001 for both). Together, these findings suggest that the subjects’ strategy consisted of aiming at a general area depending on the target broad location (left, center, or right), and then refined their endpoint position in the adjustment phase.

### Effective adjustment time

We assessed the time subjects spent in the adjustment phase before they stopped making corrections as a function of trial uncertainty (Low, High) and absolute perturbation size (0, 0.5, 1.5). The mean effective adjustment time (1.60 ± 0.06 s) was not affected by trial uncertainty per se (main effect: Low, High; *F*_(1,15)_ = 0.2, *p* = .66, $\eta_{\text{p}}^{2}=0.01$), but was significantly influenced by perturbation size (main effect: perturbation size; *F*_(1.93,28.9)_ = 20.9, *ϵ* = 0.96, *p* < .001, $\eta_{\text{p}}^{2}=0.58$) with no interaction (interaction: uncertainty × perturbation size; *F*_(2,30)_ = 0.74, *ϵ* ≈ 1, *p* > .40, $\eta_{\text{p}}^{2}=0.05$). On average, there was no difference in adjustment time between baseline and small (0.5) perturbation trials (time difference 1 ± 16 ms, *p* = .95, *d* = 0.01). However, subjects spent significantly more time adjusting their endpoint position in large (1.5) perturbation trials than baseline trials (time difference 93 ± 14 ms, paired *t*-test *t*_(15)_ = 6.89, *p* < .001, *d* = 1.72). Effective adjustment times were broadly scattered in the range 0–3 s and approximately symmetric around the mean (skewness 0.03 ± 0.08), with no sign of an accumulation near 3 s. We found qualitatively similar results by defining as ‘effective ajustment time’ the fraction of time that subjects spent moving in the adjustment phase, instead of the time elapsed before they stopped moving. Velocity profiles during the adjustment phase show that subjects performed a rapid, large correction for perturbations in perturbation trials, followed by occasional small adjustments that become less frequent with time (panel B in S1 Fig). Together, these results suggest that subjects had ample time to make the needed corrections in both Low and High uncertainty trials.

### Analysis of performance

**Fig 3:**
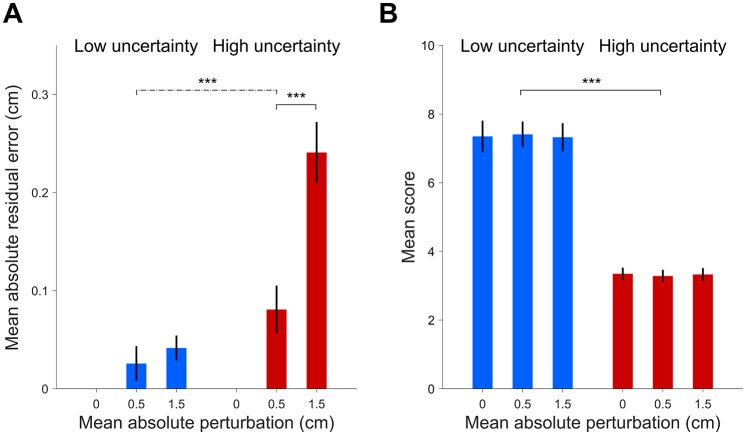
Participants’ mean absolute residual errors and mean scores. *A:* Mean absolute residual error (mean ± SE across subjects; residual errors are computed after removing the residual error for the 0 perturbation condition) by perturbation size (0, ±0.5, ±1.5 cm) and trial uncertainty (Low, High). These data are the same as in Fig 2A, here shown in absolute value and aggregated by perturbation size. *B:* Participants’ mean scores (mean ± SE between subjects) by perturbation size and trial uncertainty. Even though the residual errors (panel A) are significantly different from zero and significantly modulated by perturbation size (*p* < .001) and the interaction between the uncertainty and perturbation size (*p* < .001), the scores (panel B) are significantly affected only by the trial uncertainty (*p* < .001).

Overall, subjects showed significant mean absolute residual errors (Fig 3A) that depended on the uncertainty level (Low, High) and perturbation size (0, 0.5, 1.5). To determine how these biases affected performance, we analyzed their mean score per trial as a function of trial uncertainty and perturbation size (Fig 3B). Interestingly, the mean score was significantly influenced only by trial uncertainty (Low: 7.36 ± 0.38, High: 3.32 ± 0.14; main effect: *F*_(1,15)_ = 177, *p* < .001, $\eta_{\text{p}}^{2}=0.92$), with no significant effect of perturbation size nor interaction (*p* > 0.60 and $\eta_{\text{p}}^{2}<0.03$ for both). Analogous results hold if we split the High-uncertainty trials in left and right, depending on their location (having, thus, three levels of trial uncertainty: High-Left, Low-Middle, High-Right), and five levels of perturbations (−1.5, −0.5, 0, 0.5, 1.5), suggesting that differences are not hidden by the pooling procedure. These findings suggest that subjects’ partial lack of correction did not significantly affect their performance.

We compared subjects’ average score with that of optimal Bayesian observers (see Methods) which shared the same disks’ ratio estimation noise *σ*_*ρ*_ as the subjects but correctly computed the location of the center of mass (*D* = 2) and fully compensated for any movement error in the adjustment phase (*α* = 0). The mean score expected from the ideal observer was 9.88 ± 0.12 for Low uncertainty trials and 6.03 ± 0.26 for High uncertainty ones (mean ± SD computed via bootstrap). Overall, subjects’ average score was significantly lower (paired *t*-test *p* < .001 for both conditions), with a relative efficiency of about ∼0.75 and ∼0.55 for respectively Low and High uncertainty trials.

Our previous analysis (‘Mean residual error and variability’) showed that subjects’ corrective strategy differed between the two levels of uncertainty, with an ‘almost-full’ correction for Low uncertainty trial (∼3% uncorrected perturbation) and a ‘partial’ correction for High uncertainty trials (∼16% uncorrected perturbation). We estimated what would have been the score in the perturbed Low uncertainty conditions, had the participants adopted the partial amount of correction as in the High uncertainty trials. To estimate subjects’ score in this hypothetical case we considered their baseline, unperturbed responses and added the mean residual error from baseline, which we had previously estimated from both Low and High uncertainty trials (corresponding respectively to almost-full and partial correction). We simulated also the almost-full correction strategy as a control, expecting to observe no difference with baseline. The score in each trial was recomputed through Eq (1). The original mean score in the Low uncertainty condition, without perturbation, was 7.36 ± 0.43 (see Fig 3B). As expected, hypothetical mean scores under the almost-full correction strategy were not significantly different from baseline (7.52 ± 0.35 and 7.40 ± 0.39, respectively for small, ±0.5, and large, ±1.5, perturbations; main effect: perturbation size, *F*_(1.96,29.4)_ = 0.88, *ϵ* = 0.84, *p* = .41, $\eta_{\text{p}}^{2}=0.06$). On the contrary, hypothetical mean scores under the partial correction strategy were significantly different from baseline (6.59 ± 0.49 and 6.41 ± 0.41; main effect: perturbation size, *F*_(1.99,29.9)_ = 16.1, *ϵ* ≈ 1, *p* < .001, $\eta_{\text{p}}^{2}=0.52$). These numbers mean that had the participants been equally sloppy in their correction strategy in the Low uncertainty trials as they were in the High uncertainty trials, the drop in score would have been statistically significant and notable (ΔScore −0.97 ± 0.18; paired *t*-test *t*_(15)_ = −6.31, *p* < .001, *d* = 1.58). Conversely, the data show that had the participants been (almost) fully correcting for perturbations in the High uncertainty trials as they were in the Low uncertainty trials, the difference in score would have been negligible (no difference in score between perturbed and unperturbed High uncertainty trials, Fig 3B). This suggests that participants’ adjustment strategy took into account task demands, even in the absence of performance feedback in perturbation trials.

### Bayesian model fit

**Fig 4:**
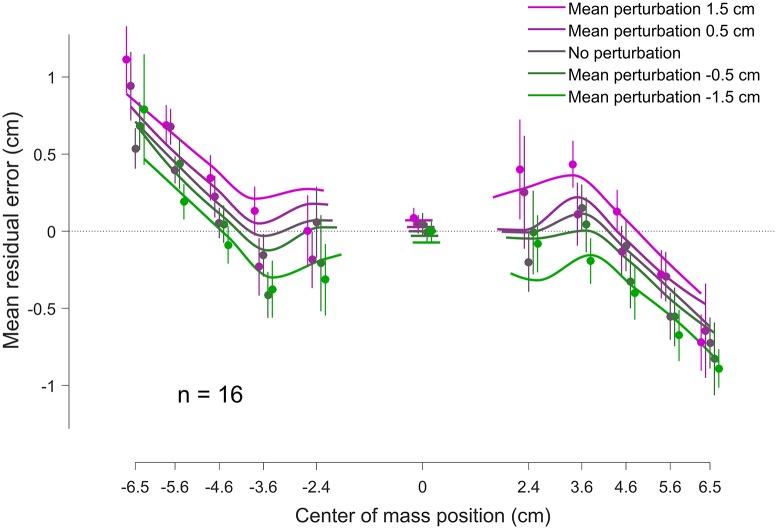
Mean residual error (bias) as a function of the location of the center of mass. Data points and error bars are mean data ± SE across subjects in the test session (binned for visualization). Colors correspond to different mean perturbation levels. Continuous lines are the fits of the Bayesian model to each individual dataset, averaged over subjects (asymmetries are due to asymmetries in the data). For both data and model fits, distinct perturbation levels are displayed with a slight offset on the *x* axis for visualization purposes. Vertical shifts in residual error for different levels of perturbation correspond to different amounts of average lack of correction (absolute residual errors shown in Fig 3A).

**Fig 5:**
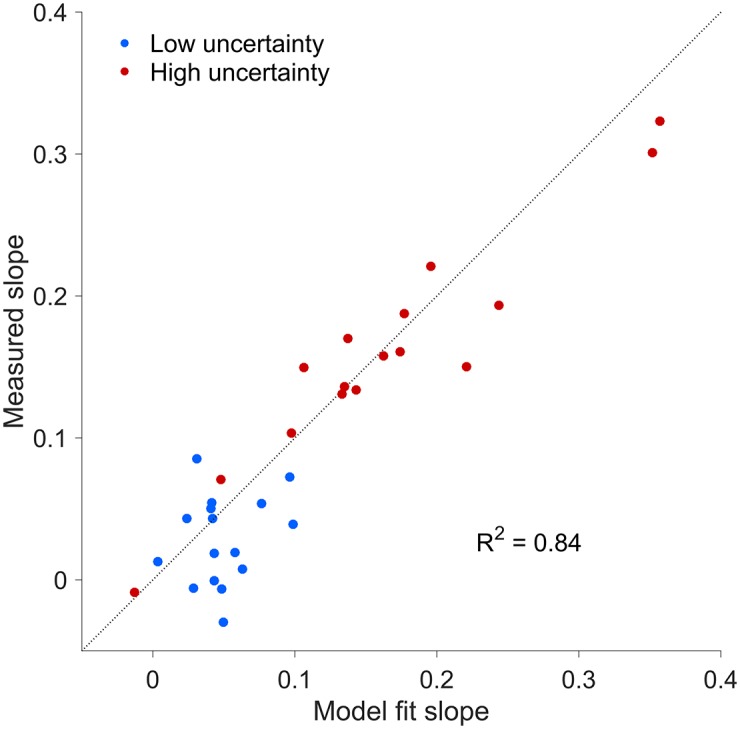
Slope of mean residual error with respect to perturbation, comparison between data and model. Each circle represents the slope of the mean residual error (Fig 2B) for a single subject for Low-uncertainty trials (blue dots) and High-uncertainty trials (red dots). The *x* axis indicates the slope predicted by the Bayesian observer model, while the *y* axis reports the slope measured from the data (slope of linear regressions in Fig 2B). The model correctly predicts the substantial difference between Low-uncertainty and High-uncertainty trials and is in good quantitative agreement with individual datasets.

We examined subjects’ mean residual errors as a function of the actual center of mass location relative to the midpoint of the bar and mean perturbation level (Fig 4). Even though individual participants’ datasets are variable, their mean residual errors exhibited a clear nonlinear pattern as a function of center of mass location, partly driven by the prior over center of mass locations (Fig 1C). We fit the Bayesian observer model to the individual datasets and obtained a good qualitative agreement with the group data (Fig 4) and quantitative agreement for the slope of mean residual error with respect to perturbation for individual subjects (*R*^2^ = 0.84; see Fig 5). Fig 4 shows that there is a separation of biases (vertical shifts) for different amount of perturbation, indicative of the influence of target uncertainty when making corrections. Moreover, we observe a regression to the means of each prior component (left and right), which stems from the shape of the prior.

A crucial element of the model is a loss function that takes into account both a final targeting error cost and an additional cost of moving in the adjustment phase. Due to the width of the posterior distribution in the High-uncertainty condition, the expected gain for an adjustment is smaller than in the Low-uncertainty condition and therefore subjects may be less willing to adjust. Our model qualitatively predicts that the lack of correction to external perturbations should correlate with the trial uncertainty (as measured by the spread of the posterior distribution).

The best fit model parameters to the data were: *σ*_*ρ*_ = 0.063 ± 0.004 (estimated from the training session), *D* = 1.94 ± 0.04 (not significantly different from the correct value *D* = 2; *t*-test *t*_(15)_ = 1.51, *p* = .15, *d* = 0.38), *σ*_motor_ = 0.76 ± 0.06 cm. Fits of the loss-related parameters showed that for 3 subjects the adjustment loss was almost constant (*σ*_*adj*_ → ∞). For the other 13 subjects we found: *α* = 3.1 ± 0.9, *σ*_adj_ = 2.8 ± 0.5 cm, suggesting that the cost changed slowly, with a large length scale (at least as large as the largest perturbations of ≈ ±2 cm), and in general these subjects were giving a sizeable weight to the adjustment term (*α* > 1; *t*-test *t*_(12)_ = 2.19, *p* = .049, *d* = 0.61). Interpreting the adjustment cost as effort, this result is in qualitative agreement with a previous study that found that effort had a considerabily greater relative weight in the loss function than the error term (relative weight ∼7 for the force production task described in the study; see [44]).

### Alternative observer models

We also analyzed the predictions of a number of alternative observer models: (1) a quadratic loss model for the error term in Eq (7); (2) a power-function loss model for the adjustment loss; (3) an alternative model which explains lack of correction as a miscalibration of the perceived position of the cursor. Alternative models (1) and (3) are unable to account for the principal effect that we observed in the experiment, that is a modulation of the amount of correction that depends on target uncertainty. We found that model (2) is empirically indistinguishable from the inverted Gaussian adjustment loss (as previoulsy reported in another context [43]), meaning that the exact shape that we posited for the adjustment loss is not critical to explain our results. In conclusion, results from these alternative observer models further validate our modelling choices. Detailed description and analysis of these alternative observer models can be found in S1 Appendix.

## Discussion

We used a task in which we could control the uncertainty of the location of a target (the center of mass) and examine the extent to which subjects corrected for perturbations of their reach to indicate the target location. We found that target uncertainty significantly affected subjects’ error correction strategy for perturbations of the visual feedback on a trial-by-trial basis, but in such a way that the overall performance would not be hindered. That is, subjects almost fully corrected for the perturbation when target uncertainty was low but only partially corrected when the target uncertainty was high.

### Effect of uncertainty on reaching behaviour

Our study differs from previous work that examines how uncertainty affects sensorimotor behavior. Studies which show that subjects can integrate priors with sensory evidence to produce optimal, yet biased, estimates are consistent with a point estimate being used by the motor system when enacting a movement [5]. The bias we show here is a bias arising from error correction which acts in addition to any biases from Bayesian integration, and would not be predicted if the motor system only had a point estimate of the target location. Moreover, the partial corrections we see relate to the posterior width within a trial. This is in contrast with studies which show that the distribution of perturbations can affect the corrections seen from one trial to the next [45].

Qualitatively similar trial-to-trial, context-dependent responses to perturbations have been observed when people are required to reach to spatially extended target [39]. In that case, corrections happened during fast reaching movements and were compatible with external task demands: errors along the larger dimension of the targets required smaller compensations to still successfully hit the targets (according to the principle of minimal intervention). In our experiment, however, subjects were sensitive to the implicit posterior width, as opposed to explicit visual target width. Optimal feedback control predicts that, under time constraints, subjects should fail to fully correct for errors that arise late in a movement due to additional requirements of endpoint stability as well as temporal limitations, even if there is no target uncertainty [28]. However, our bias is unrelated to time constraints or requirements of stability as a 3 second adjustment time ensures that sensory delays cannot prevent corrections [29], and our data show that subjects had ample time to correct for mistakes up to their desired precision. Also, note that our use of a long, fixed adjustment time window prevented decision strategies that are available if subjects can choose when to end the adjustment period and move to the next trial, thereby choosing to skip the more difficult trials [46].

An interaction between target uncertainty and response bias has been previously reported in motor planning by Grau-Moya *et al.* [34]. In their task subjects were required to hit a visual target whose horizontal location uncertainty was manipulated. A robotic interface was used to generate a resistive force that opposed motion in the outward direction with the force linearly related to the horizontal location of the hand. They found that on higher uncertainty trials subjects chose to err on the side of the target with the lower resistive force. There are several key differences of this previous study to ours. In their study, the ‘effort’ cost is explicit and externally imposed, hit/miss performance feedback is provided on all trials, and explicit manipulations of the cost are blocked by session. By contrast, here we showed an implicit, unconscious trade-off between accuracy and effort in online error correction during a naturalistic task. Moreover, in our study task-relevant perturbations (i.e., implicit manipulations of the cost) were unbeknownst to the subjects and intermixed on a trial-by-trial basis, and we did not provide performance feedback on perturbed trials. Critically, their work does not address correction to ongoing motor commands and shows that subjects can pre-plan a trade-off whereas we show that the online error correction is affected by target uncertainty. Our work provides, therefore, a stronger test of the interaction between uncertainty in the estimate and feedback control.

Finally, target uncertainty in our experiment emerged primarily from a complex mapping from stimulus to target location in a naturalistic task (calculation of the center of mass of a visual dumbbell). This type of uncertainty may differ from uncertainty arising purely from sensory estimation noise, such as with visually blurred targets [29]. On the other hand, ‘computational’ uncertainty is a common component of everyday problems the motor system needs to deal with, such as with object manipulation (see [47] for a review of different types of sensorimotor uncertainty). In our modelling, for convenience we grouped all sources of noise under the labels of ‘sensory’ (input) and ‘motor’ (output) noise but other components may well be present. Our analysis applies here irrespective of the exact nature of uncertainty in target location.

### Uncertainty and lack of correction

A somewhat surprising finding is that subjects did not fully correct for the perturbations, but in a way that did not significantly affect performance. Clearly, a null effect on score differences might simply be due to lack of statistical power in our analysis, but we demonstrated that had subjects used the same partial correction strategy in all trials, their performance would have dropped by almost one point on average. This means that subjects’ correction strategy for Low and High uncertainty trials was well adapted to task demands.

A similar finding of partial, yet ‘optimal’, correction has been reported in a recent study by van Dam and Ernst [48], that looked at subjects’ awareness of their own pointing errors. Participants performed a reaching movement to a one-dimensional target, and visual feedback of both the hand and target position was withheld after the commencement of the movement. After movement termination, subjects responded in a 2AFC task whether they had landed to the left or to the right of the target. In the condition that is most related to our work, subjects were also allowed to correct for their natural pointing mistakes, with no time limit. Also, at this point subjects would receive a brief visual feedback (with small or large blur) about their current endpoint position. The study reports that subjects hardly corrected for their mistakes, but analysis showed that the applied correction gains were sensible (if not ‘optimal’) when taking into account the information subjects had about their own pointing errors and their current endpoint position [48].

Our study differs from the work by van Dam and Ernst in several fundamental aspects. Most importantly, their work probes a form of Bayesian integration between (a) the current knowledge of endpoint position or, equivalently, estimated distance from the target (due to proprioception and provided noisy visual feedback) and (b) the prior knowledge of the error distribution (and target position). One of their main findings is that subjects seem to acquire more detailed information of the endpoint position only after the end of the movement, even for slow reaches [48]. We showed instead that in our task the lack of correction cannot be explained by a simple form of Bayesian integration. Even if subjects integrated visual feedback of the cursor with (conflicting) proprioceptive information, the expected biases would not yield the observed pattern of uncertainty-dependent corrections.

### The cost of effort and alternative explanations

Our data are consistent with an additional term in the loss function that can be interpreted as ‘effort’ (whether energy, time or computation; see [15, 21, 44]). The exact nature of this cost is left open, as our experiment does not allow us to pinpoint the specific cost. Our model provides good fits to the subjects’ data, and, moreover, we showed that other common models of loss used in Bayesian estimation and motor planning, which either ignore the cost of adjustment or use a quadratic error loss term, fail to account for the key features of our datasets.

However, our model hinges on several assumptions, and more targeted experiments may be needed to completely rule out specific alternative explanations. For example, one assumption of the model is that the observer’s posterior distribution over target location is stable within a trial and, for instance, unaffected by the reappearance of the cursor. If subjects took the reappearance of the cursor as an independent piece of evidence, an incorrect belief update (e.g., via a Kalman filter [49]) might produce effects similar to those that we observe. Such behavior is sub-optimal and unlikely since the stimulus was always present on the screen and subjects had plenty of time after the reappearance of the cursor to adjust their endpoint. Our results are also consistent with an interpetation of subjects’ behavior as a form of risk-sensitivity [32, 34]. An interesting alternative hypothesis inspired by [48] is that subjects built an internal expectation of their average error during the trials with performance feedback, and, therefore, were less willing to correct for large perturbations that were reputed to be unlikely. This interpretation predicts, among other things, that the length scale of the adjustment cost, *σ*_adj_, should correlate with the spread of the errors made by the subject, but we did not find any evidence for this pattern in the data.

A stronger empirical test for the interaction between effort and cost would consist of a ‘Bayesian transfer’ type of task [50], in which the same observers are tested on different scoring functions, amounts of required effort [34], and training. In such a task, observers would not necessarily be able to learn any arbitrary reward function, but we expect them to at least adapt to qualitative features of the provided cost, such as skewness—as found, for example, in our previous work on sensorimotor timing [7]. Arguably, the performance (and ‘optimal laziness’) will be correlated to the amount of training and inversely related to the complexity of the provided cost.

In conclusion, our results show that even for simple, naturalistic tasks such as center of mass estimation, the inertia against additional correction can be noticeable and is significantly modulated by trial uncertainty. At the same time, somewhat paradoxically, the effects on performance of this lack of correction are negligible, suggesting that subjects’ may have been ‘optimally lazy’ in correcting for their mistakes, according to the minimal intervention principle [21, 23], even in the absence of performance feedback. Our findings suggest that there is no clear-cut separation between the decision making and motor component of a task, since perceptual or cognitive uncertainty affects subsequent motor behavior beyond action initiation, as the posterior distribution is used even in the adjustment period.

## Supporting Information

S1 Fig Trajectory and velocity profiles. Full movement trajectory (*A*) and velocity profiles in the adjustment phase (*B*) for a representative subject, for respectively High-Left uncertanty (left), Low uncertainty (middle), and High-Right uncertainty (right) targets. Thick lines are mean trajectories and velocity profiles, thin lines are individual trials (subsampled for visualization). Different colors correspond to different mean perturbation levels (we show here only -1.5, 0, and 1.5 cm). *A:* Full movement trajectories. For visualization, we removed from the *x* position the random jitter of the dumbbell (linearly from *y* = 0 to *y* = 19 cm). *B:* Velocity profiles along the *x* axis during the 3 s adjustment phase. Subjects quickly reacted to the perturbation in perturbed trials, and then performed minor adjustments.
(PDF)

S1 Appendix Additional analyses and models. Extended definitions and derivations: posterior over center of mass; posterior distribution of estimated center of mass; optimal target after adjustment with inverted Gaussian loss. Model fitting. Alternative observer models: quadratic error loss; power-law adjustment loss; miscalibrated observer model. Debriefing questionnaire.
(PDF)

S1 Dataset Subjects’ datasets. MATLAB (MathWorks) file containing all sixteen subjects’ datasets for training and test session.
(MAT)
