# Supplementary figures and images for: Target Uncertainty Mediates Sensorimotor Error Correction
Source: PLoS One. 2017 Jan 27;12(1):e0170466. doi: 10.1371/journal.pone.0170466 (PMC5271325; doi:10.1371/journal.pone.0170466)

High uncertainty (left)

Low uncertainty

High uncertainty (right)

**A**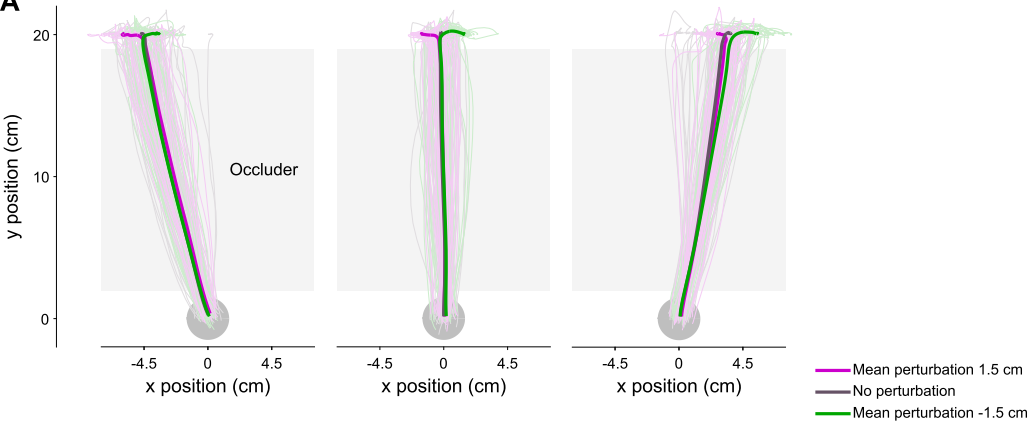**B**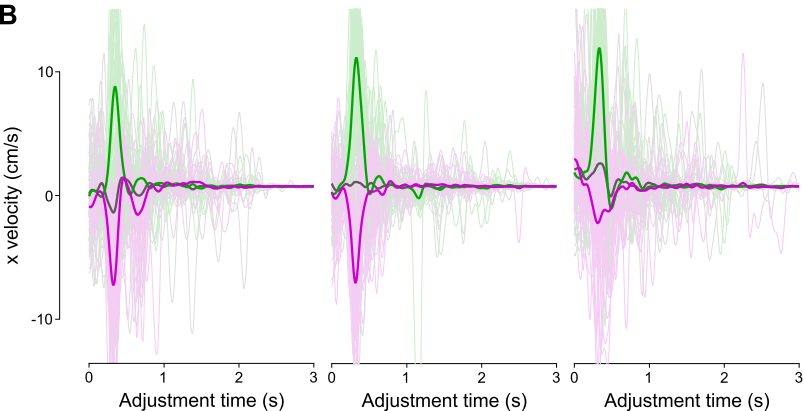

Supplement: S1 Fig — Full movement trajectory (A) and velocity profiles in the adjustment phase (B) for a representative subject, for respectively High-Left uncertanty (left), Low uncertainty (middle), and High-Right uncertainty (right) targets. Thick lines are mean trajectories and velocity profiles, thin lines are individual trials (subsampled for visualization). Different colors correspond to different mean perturbation levels (we show here only -1.5, 0, and 1.5 cm). A: Full movement trajectories. For visualization, we removed from the x position the random jitter of the dumbbell (linearly from y = 0 to y = 19 cm). B: Velocity profiles along the x axis during the 3 s adjustment phase. Subjects quickly reacted to the perturbation in perturbed trials, and then performed minor adjustments. (PDF) [file pone.0170466.s001.pdf]
