# Supplementary material for: Target Uncertainty Mediates Sensorimotor Error Correction
Source: PLoS One. 2017 Jan 27;12(1):e0170466. doi: 10.1371/journal.pone.0170466 (PMC5271325; doi:10.1371/journal.pone.0170466)
Supplement: S1 Appendix — Extended definitions and derivations: posterior over center of mass; posterior distribution of estimated center of mass; optimal target after adjustment with inverted Gaussian loss. Model fitting. Alternative observer models: quadratic error loss; power-law adjustment loss; miscalibrated observer model. Debriefing questionnaire. (PDF) [file pone.0170466.s002.pdf]

Target Uncertainty Mediates Sensorimotor Error Correction
Supporting Information: Appendix S1
Additional analyses and models

Luigi Acerbi, Sethu Vijayakumar and Daniel M. Wolpert

Contents

1 Extended definitions and derivations **1**
1.1 Posterior over center of mass . . . 1
1.2 Posterior distribution of estimated center of mass . . . 2
1.3 Optimal target after adjustment with inverted Gaussian loss . . . 3

2 Model fitting **3**

3 Alternative observer models **4**
3.1 Quadratic error loss . . . 4
3.2 Power-law adjustment loss . . . 4
3.3 Miscalibrated observer model . . . 4

4 Debriefing questionnaire **6**

Supplemental References **6**

1 Extended definitions and derivations

1.1 Posterior over center of mass

The posterior over center of mass is represented by Eq. 4 in the main text, where we defined the mixing weights, means and variances as:

$$Z^{(i)} \equiv \mathcal{N}\left(\rho_m \left| \mu^{(i)}_{\text{prior}}, {\sigma^{(i)}_{\text{prior}}}^2 + \sigma^2_\rho \right.\right) \quad \text{and} \quad \mathcal{Z} \equiv \sum_{i=1}^{3} Z^{(i)} \quad \text{(normalization constant)},$$

$$\mu^{(i)}_{\text{post}} \equiv \frac{\rho_m {\sigma^{(i)}_{\text{prior}}}^2 + \mu^{(i)}_{\text{prior}} \sigma^2_\rho}{{\sigma^{(i)}_{\text{prior}}}^2 + \sigma^2_\rho}, \qquad \sigma^2_{\text{post}} \equiv \frac{{\sigma^{(i)}_{\text{prior}}}^2 \sigma^2_\rho}{{\sigma^{(i)}_{\text{prior}}}^2 + \sigma^2_\rho}, \qquad \text{for } i = 1, 2, 3,$$

where ρ_m is the noisy measurement of log disks ratio, σ^2_ρ the noise variance, and $\mu^{(i)}_{\text{prior}}$, ${\sigma^{(i)}_{\text{prior}}}^2$ are, respectively, mean and variance of the i-th mixture component of the prior over log disks ratios (for $i = 1, 2, 3$).

1.2 Posterior distribution of estimated center of mass

The horizontal location of the center of mass s of two D-dimensional spheres with radii r_1 and r_2, separated by a distance ℓ, is given by:

$$s = \frac{\ell}{2} \cdot \left(\frac{r_2^D - r_1^D}{r_1^D + r_2^D} \right), \tag{S1}$$

with respect to the midpoint of the target line. From Eq. S1, the relationship between the log ratio of the radii of two spherical objects in a D-dimensional space and their center of mass, s, can be represented by the mapping f_D:

$$f_D(\rho) = \frac{\ell}{2} \cdot \left[\frac{(-1) \cdot r_1^D + (+1) \cdot r_2^D}{r_1^D + r_2^D} \right] = \frac{\ell}{2} \cdot \left[\frac{\frac{r_2^D}{r_1^D} - 1}{\frac{r_2^D}{r_1^D} + 1} \right] = \frac{\ell}{2} \cdot \left[\frac{e^{D\rho} - 1}{e^{D\rho} + 1} \right], \tag{S2}$$

whose inverse is $f_D^{-1}(s) = \frac{1}{D} \log \left[(\ell/2 + s)/(\ell/2 - s) \right]$. We assume that the observer uses the mapping f_D from Eq. S2 with some fixed value of $D > 0$, although not necessarily the correct value $D = 2$ for two-dimensional disks, nor we restrict D to be an integer.

Combining Eqs. 4 of the main text with Eq. S2, the posterior distribution of the location of the estimated center of mass takes the form:

$$\begin{aligned} q_{\text{post}}(s|\rho_m) &= \int_{-\infty}^{\infty} \delta \left[f_D(\rho) - s \right] q_{\text{post}}(\rho|\rho_m) d\rho \\ &= \frac{1}{\mathcal{Z}} \sum_{i=1}^{3} Z^{(i)} \int_{-\infty}^{\infty} \delta \left(\rho' - s \right) \mathcal{N} \left(f_D^{-1}(\rho') \left| \mu_{\text{post}}^{(i)}, {\sigma_{\text{post}}^{(i)}}^2 \right. \right) \frac{d\rho'}{\frac{df_D}{d\rho} \left(f^{-1}(\rho') \right)} \\ &= \frac{1}{\mathcal{Z}} \sum_{i=1}^{3} Z^{(i)} \mathcal{N} \left(\frac{1}{D} \log \left[\frac{\ell/2 + s}{\ell/2 - s} \right] \left| \mu_{\text{post}}^{(i)}, {\sigma_{\text{post}}^{(i)}}^2 \right. \right) \frac{\ell}{D \cdot (\ell^2/4 - s^2)}, \end{aligned} \tag{S3}$$

where in the second passage we have performed the change of variable $\rho' = f_D(\rho)$.

The posterior in Eq. S3 is not a mixture of Gaussian distributions due to the nonlinear relationship in the argument of the Gaussian function. To allow efficient computation of optimal behavior we can approximate each mixture component with a Gaussian:

$$q_{\text{post}}(s|\rho_m) \approx \frac{1}{\mathcal{Z}} \sum_{i=1}^{3} Z^{(i)} \mathcal{N} \left(s \left| m_{\text{post}}^{(i)}, {s_{\text{post}}^{(i)}}^2 \right. \right), \tag{S4}$$

where $m_{\text{post}}^{(i)}$ and $s_{\text{post}}^{(i)}$ are respectively the mean and SD of the mixture components in Eq. S3 (computed numerically, function `trapz` in MATLAB).

The 'Gaussianized' components in Eq. S4 are a very good approximation of their counterparts in Eq. S3 for the parameters in our task, as measured by an average Kullback-Leibler divergence of $(3.1 \pm 0.7) \cdot 10^{-3}$ nats (mean $\pm$ SD across all posteriors in the task). This amounts to roughly the KL divergence between two Gaussians with same variance and whose difference in means is one-twelfth of their SD. The approximation we chose works better than a Laplace approximation [1], which yields worse values for the KL divergence of $(7.8 \pm 1.8) \cdot 10^{-3}$ nats.

1.3 Optimal target after adjustment with inverted Gaussian loss

Combining Eqs. 5 and 8 of the main text with inverted Gaussian loss functions for both error and adjustment,

$$\mathcal{L}_{\text{err}}(\hat{s}-s) = -\exp\left\{-\frac{(\hat{s}-s)^2}{2\sigma_{\text{err}}^2}\right\}, \qquad \mathcal{L}_{\text{adj}}(\hat{r}-r) = -\exp\left\{-\frac{(\hat{r}-r)^2}{2\sigma_{\text{adj}}^2}\right\},$$

we obtain the final expression for the 'optimal' target after adjustment,

$$\begin{aligned} s^*(\rho_m,\tilde{r}) &= \arg\min_{\hat{s}}\left[\alpha\mathcal{L}_{\text{adj}}(\hat{s}-\tilde{r}) + \int_{-\ell/2}^{\ell/2} q_{\text{post}}(s|\rho_m)\mathcal{L}_{\text{err}}(\hat{s}-s)\,ds\right] \\ &= \arg\min_{\hat{s}}\left[-\alpha\sqrt{2\pi}\sigma_{\text{adj}}\mathcal{N}\left(\hat{s}\left|\tilde{r},\sigma_{\text{adj}}^2\right.\right) - \sqrt{2\pi}\sigma_{\text{err}}\int_{-\ell/2}^{\ell/2} q_{\text{post}}(s|\rho_m)\mathcal{N}\left(s\left|\hat{s},\sigma_{\text{err}}^2\right.\right)ds\right] \\ &= \arg\min_{\hat{s}}\left[-\widetilde{\alpha}\mathcal{N}\left(\hat{s}\left|\tilde{r},\sigma_{\text{adj}}^2\right.\right) - \sum_{i=1}^{3} Z^{(i)}\mathcal{N}\left(\hat{s}\left|m_{\text{post}}^{(i)}, {s_{\text{post}}^{(i)}}^2 + \sigma_{\text{err}}^2\right.\right)\right], \end{aligned} \tag{S5}$$

where we have collected all scaling factors in $\widetilde{\alpha} \equiv \alpha\mathcal{Z}\sigma_{\text{adj}}/\sigma_{\text{err}}$, by using the property that the arg min is invariant to rescaling of its argument by a constant.

In order to find s_{pre}^* (Eq. 6 in the main text) and subsequently the 'optimal' final position s^* (Eq. S5), we used `gmm1max`, a fast numerical algorithm for finding the mode of a mixture of Gaussians [2,3], implemented in the `gmm1` package for MATLAB (`https://github.com/lacerbi/gmm1`), as both equations have no known analytical solution.

2 Model fitting

Using Eq. 9 in the main text for the probability of response in a given trial, we estimated the model parameters for each subject by maximizing the (log) likelihood of the data:

$$\log\mathcal{L}(\boldsymbol{\theta}) = \sum_{i=1}^{N}\log\left[\Pr{}^*\left(r^{(i)}|\rho^{(i)},b^{(i)};\boldsymbol{\theta}\right)\right], \tag{S6}$$

where $N = 576$ is the total number of trials and we have assumed conditional independence between trials. We modified Eq. 9 in the main text with a tiny probability ϵ to improve robustness of the inference:

$$\Pr{}^*(r|\rho,b;\boldsymbol{\theta}) = (1-\epsilon)\Pr(r|\rho,b;\boldsymbol{\theta}) + \epsilon, \tag{S7}$$

where $\epsilon = 1.5\cdot 10^{-5}$ is the value of the pdf of a normal distribution five SDs away from the mean. Eq. S7 allows for a very small lapse rate that prevents a few outliers in a subject's dataset from having a large effect on the log likelihood of the data in Eq. S6.

Maximum likelihood fits were obtained via the Nelder-Mead simplex method (`fminsearch` in MATLAB), with ten randomized starting points per dataset. The log likelihood landscapes proved to be relatively simple, with almost all optimization runs converging to the same optimum within each dataset.

3 Alternative observer models

3.1 Quadratic error loss

As an alternative model of loss for the error term in Eq. 7 of the main text, we considered a quadratic loss function: $\mathcal{L}_{\text{quad}}(r - s) = (r - s)^2$. In this case, the 'optimal' target (Eq. 8 in the main text, and see also Eq. S5 for comparison) takes the form:

$$\begin{aligned} s^*(\rho_m, x_0) &= \arg\min_{\hat{s}} \left[\alpha \mathcal{L}_{\text{adj}}(\hat{s} - \tilde{r}) + \int_{-\ell/2}^{\ell/2} q_{\text{post}}(s|\rho_m) \left(\hat{s} - s\right)^2 ds \right] \\ &= \arg\min_{\hat{s}} \left[\alpha \mathcal{L}_{\text{adj}}(\hat{s} - \tilde{r}) + \hat{s}^2 - 2\hat{s} \cdot m_{\text{post}} \right], \end{aligned} \tag{S8}$$

where m_{post} is the mean of the full posterior in Eqs. S3 and S4. For example, for a quadratic adjustment loss, Eq. S8 reduces to a simple analytical solution: $s^*(\rho_m, \tilde{r}) = (m_{\text{post}} + \alpha\tilde{r})/(1 + \alpha)$. However, for *any* shape of the adjustment loss, Eq. S8 predicts that the optimal target, and therefore the amount of correction to a perturbation, does *not* depend on the variance (i. e., on the uncertainty) of the posterior distribution [4]. This prediction is at odds with the patterns observed in the experiment, so the quadratic loss model of the error is unable to account for the key feature of our data.

3.2 Power-law adjustment loss

To explore alternative models of adjustment cost in Eq. 7 of the main text, we assume an inverted Gaussian for the error combined with a power function for the adjustment loss. A power function with power $\nu > 0$ includes several common models of loss as specific cases (absolute, sub-quadratic, quadratic) and therefore represents a valid alternative to the inverted Gaussian [5]. For this case, the 'optimal' target is:

$$s^*(\rho_m, x_0) = \arg\min_{\hat{s}} \left[\frac{\alpha \mathcal{Z}}{\sigma_{\text{err}}} \left|\hat{s} - \tilde{r}\right|^{\nu} - \sum_{i=1}^{3} Z^{(i)} \mathcal{N}\left(\hat{s} \left| m_{\text{post}}^{(i)}, {s_{\text{post}}^{(i)}}^2 + \sigma_{\text{err}}^2 \right.\right) \right]. \tag{S9}$$

We compared the performances of the observer models with power loss and inverted Gaussian loss in terms of (log) maximum likelihood. We do not need to consider a term for complexity as the two models have the same number of parameters. The performance of the two models was largely similar, with a nonsignificant advantage of the inverted Gaussian model (0.7 ± 1.6 difference in average log likelihood; t-test $t_{(15)} = 0.44$, $p > .60$, $d = 0.11$). An analogous lack of empirical difference between the inverted Gaussian loss and the power loss was reported in a previous study [5]. The choice between the two functions must therefore be driven by other considerations, such as mathematical tractability (e. g., in our case the inverted Gaussian loss allows to write the expected loss in closed form, see Eq. S5).

3.3 Miscalibrated observer model

Finally, we examined the predictions for an observer model built from a different set of assumptions. For this model, we hypothesized that the source of the lack of correction is not effort, but a miscalibration of the perceived position of the cursor. Even though in our task the visual feedback of the cursor location during the adjustment phase is unambiguous, subject's perception may be systematically altered by proprioceptive feedback according to the relative reliability of vision and proprioception (see [6, 7]). In particular, the posterior distribution of cursor position r for visual cue x_{vis} and proprioceptive cue position

$x_{\rm prop}$ is given by [8]:

$$
\begin{aligned}
q_{\rm cursor}\left(r\left|x_{\rm vis},x_{\rm prop}\right.\right) &= \mathcal{N}\left(r\left|x_{\rm vis},\sigma^2_{\rm vis}\right.\right)\mathcal{N}\left(r\left|x_{\rm prop},\sigma^2_{\rm prop}\right.\right) \\
&\propto \mathcal{N}\left(r\left|\frac{x_{\rm vis}\sigma^2_{\rm prop}+x_{\rm prop}\sigma^2_{\rm vis}}{\sigma^2_{\rm vis}+\sigma^2_{\rm prop}},\frac{\sigma^2_{\rm vis}\sigma^2_{\rm prop}}{\sigma^2_{\rm vis}+\sigma^2_{\rm prop}}\right.\right),
\end{aligned}
\tag{S10}
$$

where $\sigma^2_{\rm vis}$ and $\sigma^2_{\rm prop}$ are the (subjective) variances of visual and proprioceptive noise. Noting that in our setup $x_{\rm prop} \approx x_{\rm vis} - b$, where b is the visual perturbation applied in the trial, we can rewrite Eq. S10 as:

$$
\begin{aligned}
q_{\rm cursor}\left(r\left|x_{\rm vis},x_{\rm prop}\right.\right) &\propto \mathcal{N}\left(r\left|x_{\rm vis}-b\frac{\sigma^2_{\rm vis}}{\sigma^2_{\rm vis}+\sigma^2_{\rm prop}},\frac{\sigma^2_{\rm vis}\sigma^2_{\rm prop}}{\sigma^2_{\rm vis}+\sigma^2_{\rm prop}}\right.\right) \\
&\equiv \mathcal{N}\left(r\left|x_{\rm vis}-\mu_{\rm cursor},\sigma^2_{\rm cursor}\right.\right),
\end{aligned}
\tag{S11}
$$

where we have defined $\mu_{\rm cursor}$ and $\sigma_{\rm cursor}$ for notational convenience. We assume now that, according to BDT, the observer places the visual cursor so as to match the 'optimal' visual cursor location $x^*_{\rm vis}$ that minimizes the expected loss of the task:

$$
\begin{aligned}
x^*_{\rm vis}(\rho_m) &= \arg\min_{\hat{x}_{\rm vis}}\left[-\int_{-\ell/2}^{\ell/2} q_{\rm post}(s|\rho_m)\mathcal{N}\left(r\left|\hat{x}_{\rm vis}-\mu_{\rm cursor},\sigma^2_{\rm cursor}\right.\right)\mathcal{N}\left(r\left|s,\sigma^2_{\rm score}\right.\right)ds dr\right] \\
&= \arg\min_{\hat{x}_{\rm vis}}\left[-\int_{-\ell/2}^{\ell/2} q_{\rm post}(s|\rho_m)\mathcal{N}\left(s\left|\hat{x}_{\rm vis}-\mu_{\rm cursor},\sigma^2_{\rm cursor}+\sigma^2_{\rm score}\right.\right)ds\right],
\end{aligned}
\tag{S12}
$$

where the subjective error in the loss function is computed with respect to the unknown exact cursor position r, whose distribution is inferred via cue combination (Eq. S11). The posterior over target locations, $q_{\rm post}\left(s\left|\rho_m\right.\right)$, is defined by the mixture of Gaussians of Eq. S4. For the sake of argument, let us consider the case in which the posterior is mainly represented by a single Gaussian component with mean $m_{\rm post}$ and variance $s^2_{\rm post}$ (both depend on ρ_m). This is a reasonable approximation in most of the trials as our observers' sensory noise on the estimation of the disks' (log) ratio, σ_ρ, is much smaller than the separation between components of the prior over (log) ratios ($0.063 \ll 0.405$, see main text). Using a single Gaussian in Eq. S12 we obtain:

$$
\begin{aligned}
x^*_{\rm vis}(\rho_m) =&\ \arg\min_{\hat{x}_{\rm vis}}\left[-\int_{-\ell/2}^{\ell/2}\mathcal{N}\left(s\left|m_{\rm post},s^2_{\rm post}\right.\right)\mathcal{N}\left(s\left|\hat{x}_{\rm vis}-\mu_{\rm cursor},\sigma^2_{\rm cursor}+\sigma^2_{\rm score}\right.\right)ds\right] \\
=&\ \arg\min_{\hat{x}_{\rm vis}}\left[-\mathcal{N}\left(\hat{x}_{\rm vis}\left|m_{\rm post}+\mu_{\rm cursor},\sigma^2_{\rm cursor}+\sigma^2_{\rm score}+s^2_{\rm post}\right.\right)\right] \\
=&\ m_{\rm post}+b\frac{\sigma^2_{\rm vis}}{\sigma^2_{\rm vis}+\sigma^2_{\rm prop}},
\end{aligned}
\tag{S13}
$$

where we used the definition of $\mu_{\rm cursor}$ from Eq. S11. Similarly to the quadratic error loss model, the solution in Eq. S13 does *not* depend on the variance of the posterior $s^2_{\rm post}$, meaning that for the majority of trials the observer model does not present the features that we observe in our data. Still, for trials with multimodal posteriors, the solution of Eq. S12 *might* depend on the uncertainty in the trial. If so, albeit unlikely, these trials alone might be enough to produce the effect that we observe in our data. We, therefore, analyzed the behavior that this observer model would predict for our observers for a range of reasonable values of the parameters $\sigma_{\rm vis}$ and $\sigma_{\rm prop}$ from 0.1 cm to 2.5 cm [6]. For all combinations of parameters and for all subjects we did not find any sign of interaction between trial uncertainty and residual error, confirming the results of our first approximation (Eq. S13). In conclusion, subjects' perception of the perturbed cursor position may be altered by proprioceptive feedback, but this effect alone cannot account for the uncertainty-dependent modulation of the residual error.

4 Debriefing questionnaire

Participants were presented with a short debriefing questionnaire at the end of the test session. The questionnaire asked:

1. *Did you notice any difference between trials in which you received performance feedback and trials in which you did not receive performance feedback?* [Yes / No]

2. *Did you notice any discrepancy between location of the cursor and position of your fingertip in one or more trials during this session?* [Yes / No]

If the answer was 'Yes' to any question, subjects could write a short explanation. Out of 16 subjects, one did not fill in the questionnaire; 13 subjects answered 'No' to the 1st question; and 15 subjects answered 'No' to the 2nd question. One of the participants who had answered 'Yes' to the 1st question wrote that "*After two/three trials with feedback it was harder*". Participants reacted with surprise when they were explained the experimental manipulation, after they had completed the questionnaire. Overall, we take these results as evidence that subjects were unaware of cursor displacement or of any other difference between trials with or without performance feedback.

Supplemental References

1. MacKay DJ. Information theory, inference and learning algorithms. Cambridge University Press; 2003.

2. Carreira-Perpiñán MÁ. Mode-finding for mixtures of Gaussian distributions. IEEE T Pattern Anal. 2000;22(11):1318–1323.

3. Acerbi L, Vijayakumar S, Wolpert DM. On the Origins of Suboptimality in Human Probabilistic Inference. PLoS Comput Biol. 2014;10(6):e1003661.

4. Grau-Moya J, Ortega PA, Braun DA. Risk-sensitivity in Bayesian sensorimotor integration. PLoS Comput Biol. 2012;8(9):e1002698.

5. Körding KP, Wolpert DM. The loss function of sensorimotor learning. Proc Natl Acad Sci USA. 2004;101(26):9839–9842.

6. van Beers RJ, Sittig AC, van der Gon JJD. How humans combine simultaneous proprioceptive and visual position information. Exp Brain Res. 1996;111(2):253–261.

7. van Beers RJ, Sittig AC, van der Gon JJD. Integration of proprioceptive and visual position-information: An experimentally supported model. J Neurophysiol. 1999;81(3):1355–1364.

8. Ernst MO, Banks MS. Humans integrate visual and haptic information in a statistically optimal fashion. Nature. 2002;415(6870):429–433.
